# Renal Artery Stenosis Alters Gene Expression in Swine Scattered Tubular-Like Cells

**DOI:** 10.3390/ijms20205069

**Published:** 2019-10-12

**Authors:** Arash Aghajani Nargesi, Xiang-Yang Zhu, Yuanhang Liu, Hui Tang, Kyra L. Jordan, Lilach O. Lerman, Alfonso Eirin

**Affiliations:** 1 Department of Internal Medicine, Division of Nephrology and Hypertension, Mayo Clinic, Rochester, MN 55905, USA; arash.aan@gmail.com (A.A.N.); zhu.xiangyang@mayo.edu (X.-Y.Z.); Liu.Yuanhang@mayo.edu (Y.L.); Tang.Hui@mayo.edu (H.T.); Jordan.Kyra@mayo.edu (K.L.J.); Lerman.Lilach@mayo.edu (L.O.L.); 2 Health Sciences Research & Division of Biomedical Statistics and Informatics, Mayo Clinic, Rochester, MN 55905, USA

**Keywords:** renal artery stenosis, hypertension, mRNA sequencing, scattered tubular-like cells

## Abstract

Background: Scattered tubular-like cells (STCs) proliferate and differentiate to support neighboring injured renal tubular cells during recovery from insults. Renal artery stenosis (RAS) induces renal ischemia and hypertension and leads to loss of kidney function, but whether RAS alters renal endogenous repair mechanisms, such as STCs, remains unknown. We hypothesize that RAS in swine modifies the messenger RNA (mRNA) profile of STCs, blunting their in vitro reparative capacity. Methods: CD24+/CD133+ STCs were isolated from pig kidneys after 10-weeks of RAS or sham (*n* = 3 each) and their gene cargo analyzed using high-throughput mRNAseq. Expression profiles for upregulated and downregulated mRNAs in RAS-STCs were functionally interpreted by gene ontology analysis. STC activation was assessed by counting the total number of STCs in pig kidney sections using flow cytometry, whereas cell proliferation was assessed in vitro. Results: Of all expressed genes, 1430 genes were upregulated and 315 downregulated in RAS- versus Normal-STCs. Expression of selected candidate genes followed the same fold change directions as the mRNAseq findings. Genes upregulated in RAS-STCs were involved in cell adhesion, extracellular matrix remodeling, and kidney development, whereas those downregulated in RAS-STCs are related to cell cycle and cytoskeleton. The percentage of STCs from dissociated kidney cells was higher in RAS versus Normal pigs, but their proliferation rate was blunted. Conclusions: Renal ischemia and hypertension in swine induce changes in the mRNA profile of STCs, associated with increased STC activation and impaired proliferation. These observations suggest that RAS may alter the reparative capacity of STCs.

## 1. Introduction

Renal tubules have a limited endogenous repair capacity that is circumscribed to the replacement of cells that are lost during injury. Adult differentiated tubular epithelial cells that survive episodes of acute kidney injury (AKI) undergo a process characterized by upregulation of dedifferentiation and injury markers to become scattered tubular-like cells (STCs) [1,2]. Previous studies have shown that CD24+/CD133+ STCs possess self-renewal and multi-lineage potential and can be induced to generate mature, functional, renal tubular cells [3,4]. 

Exogenous delivery of STCs can regenerate tubular structures of different portions of the nephron and ameliorate renal dysfunction in animal models of acute and chronic renal damage. For example, administration of STCs ameliorated morphologic kidney damage in murine models of AKI [5] and promoted the recovery of renal function similar to bone marrow-derived mesenchymal stem cells [6]. Likewise, delivered STCs engrafted within the mouse kidney and regenerated injured tubular cells following AKI [4]. In agreement, our group has shown that STC-derived extracellular vesicles improved perfusion and oxygenation in the stenotic murine kidney, highlighting the importance of STCs in the endogenous reparative capacity of the kidney [7]. However, whether renal disease modifies the characteristics of endogenous STCs remains to be elucidated.

Renal artery stenosis (RAS) is an important cause of secondary hypertension and renal dysfunction that produces a reduction in renal blood flow (RBF) and chronic ischemia [8,9]. RAS activates several deleterious cascades of cellular injury, such as disruption and dissociation of the cytoskeleton, changes in intracellular calcium, activation of phospholipases, and generation of oxygen radicals, which contribute to significant tubular cell damage [10,11]. However, it is unclear if RAS interferes with renal endogenous repair mechanisms, such as STCs. In the current study, we employed a well-established preclinical model of RAS in swine and high-throughput messenger RNA sequencing (mRNAseq) to test the hypothesis that RAS modifies the genetic profile of STCs, blunting their in vitro reparative capacity. 

## 2. Results

### 2.1. Systemic Characteristics and Renal Function

Domestic pigs underwent unilateral RAS or sham and were studied in vivo 10 weeks later. Table 1 shows the systemic characteristics and renal function of Normal and RAS pigs at the end of the study. All pigs had a similar body weight. Ten weeks after induction of RAS, pigs achieved hemodynamically significant degrees of stenosis. Systolic, diastolic, and mean blood pressure were elevated in RAS compared to Normal, as were serum creatinine levels. Renal hemodynamics and function were assessed with multi-detector computed tomography (MDCT). Single-kidney cortical volume and perfusion, RBF, and glomerular filtration rate (GFR) were lower in RAS versus Normal, but medullary volume and perfusion did not differ between the groups. 

### 2.2. STC Characterization

**Figure 1:**
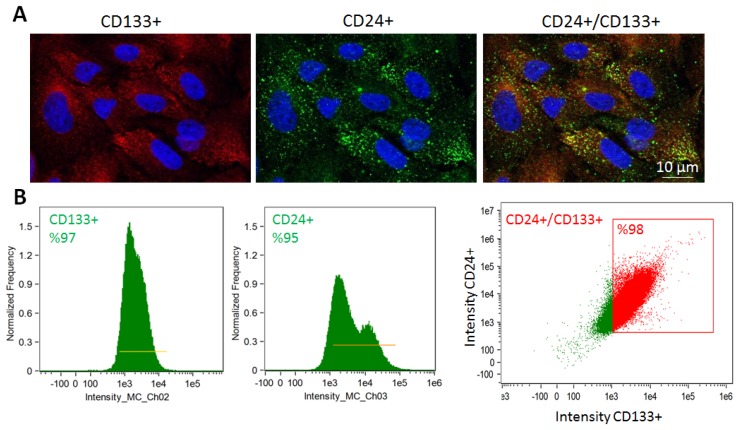
Characterization of Scattered tubular-like cells (STCs) isolated from pig kidneys. (**A**) Representative immunofluorescence staining (original magnification 40*X*) for the STC surface markers CD133 (red) and CD24 (green) in isolated swine STCs. (**B**) Flow cytometry analysis of isolated STCs showing that 97% of cells expressed CD133, 95% CD24, and 98% were double-positive for CD133 and CD24 (*n* = 3 each).

STCs were isolated from fresh pig kidneys and characterized, as previously shown [7,12]. Porcine cultured STCs co-expressed CD133 and CD24 as per both immunofluorescence staining and flow cytometry, with at a purity of 98% (Figure 1). 

### 2.3. mRNAseq Analysis

**Figure 2:**
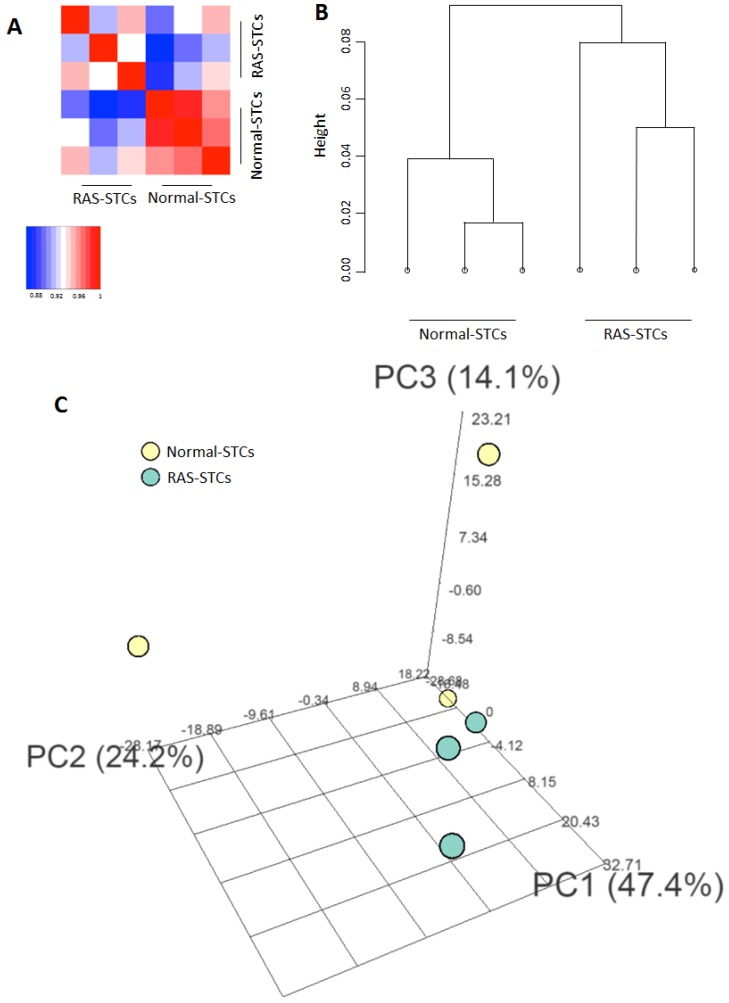
mRNAseq analysis. (**A**) Correlation between messenger RNA sequencing data derived from scattered tubular-like cells (STCs), showing well-defined Normal and renal artery stenosis (RAS) groups. (**B**) Hierarchical clustering showing the pattern of expression of Normal- and RAS-STCs (*n* = 3 each). (**C**) Three-dimensional plots of the first three components of STCs showing complete separation between samples from Normal and RAS pigs (*n* = 3 each).

The mRNAseq data of each individual sample generated in this study are available as a supplemental file in a spreadsheet format (Table S1). Mapping of mRNA reads from cultured STCs revealed a total of 11,642 expressed genes with average counts per million (CPM) > 2. Correlation plot showed well-defined Normal and RAS groups (Figure 2A). Hierarchical clustering showed that mRNA expression patterns were distinguishable between Normal- and RAS-STCs (Figure 2B), which was confirmed by principal component analysis (Figure 2C). 

### 2.4. Genes Upregulated in RAS-STCs 

**Figure 3:**
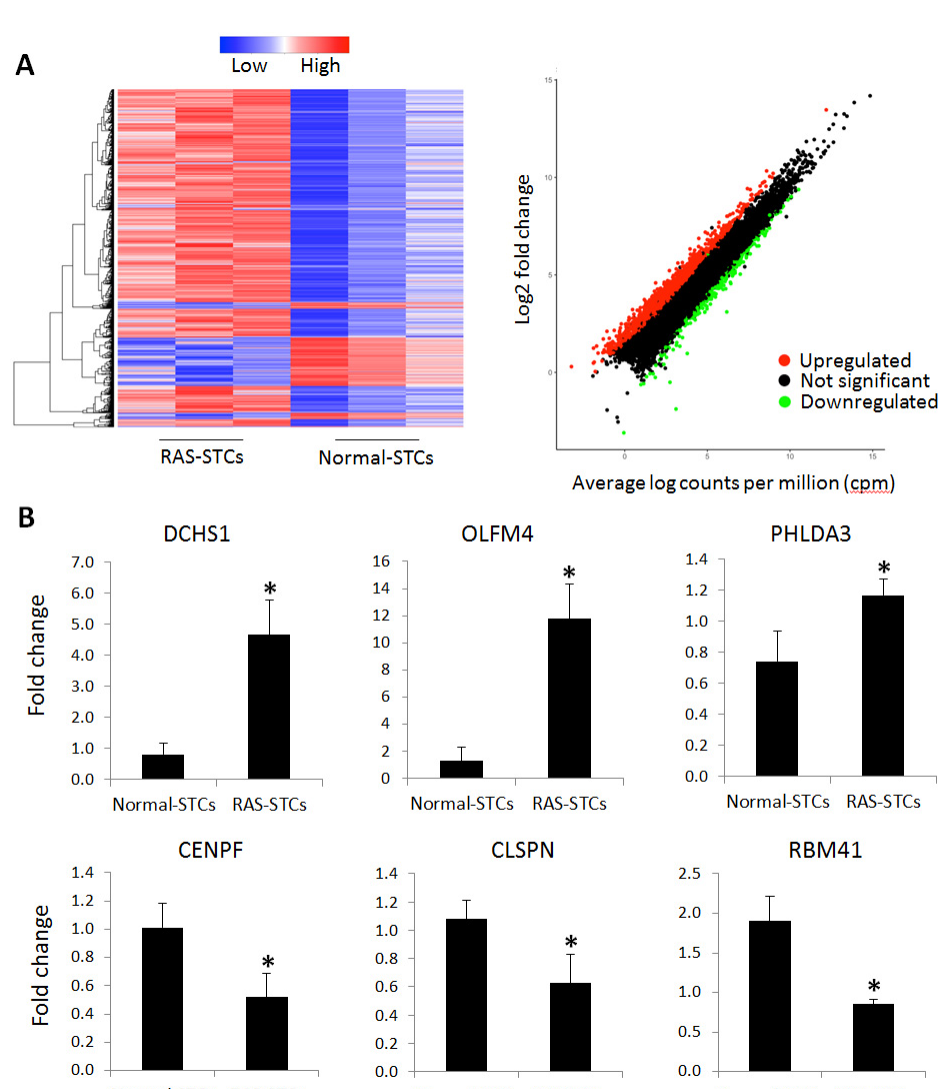
messenger RNA (mRNA) differentially expressed in renal artery stenosis (RAS)-scattered tubular-like cells (STCs). (**A**) Heat map and scatter plot showing 1,430 genes upregulated and 315 genes downregulated in RAS- versus Normal-STCs (*n* = 3 each). (**B**) Expression of selected candidate genes followed the same patterns as the mRNA sequencing findings, where Dachsous Cadherin-Related 1 (DCHS1), Olfactomedin 4 (OLFM4), and Pleckstrin Homology Like Domain Family A Member 3 (PHLDA3) were upregulated and Centromere Protein F (CENPF), Claspin (CLSPN), and RNA Binding Motif Protein 41 (RBM41) were downregulated in RAS-STCs (*n* = 3 each). * *p* < 0.05 vs. Normal-STCs.

mRNAseq analysis identified a total of 1745 genes dysregulated in RAS-STCs, among which 1,430 were upregulated and 315 downregulated compared to Normal-STCs (Figure 3A).

**Figure 4:**
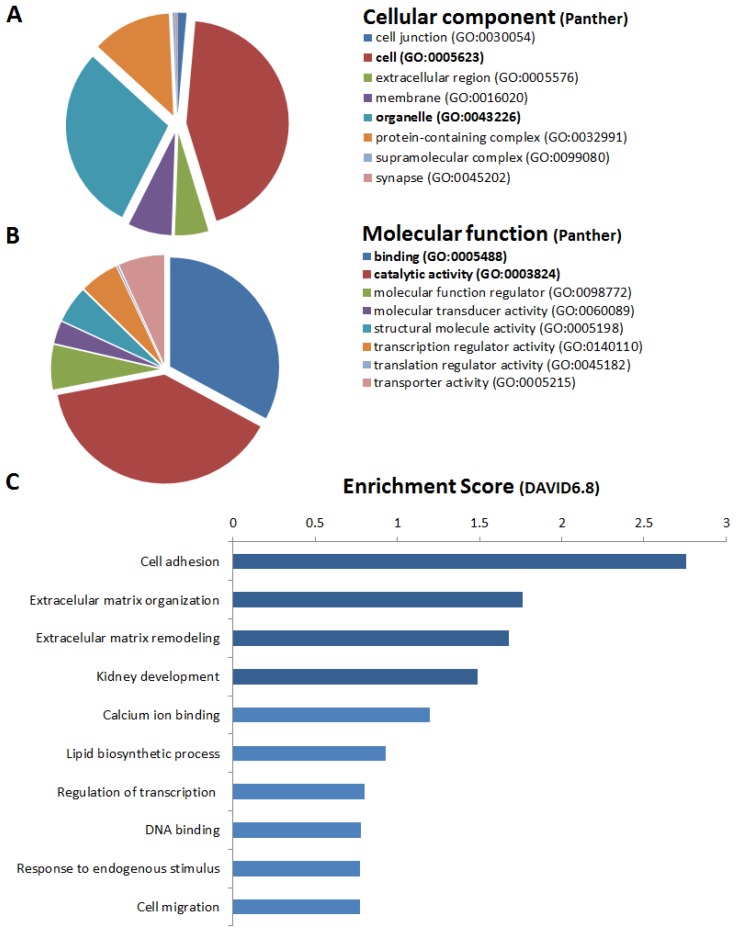
Genes upregulated in renal artery stenosis (RAS)-scattered tubular-like cells (STCs). Protein Analysis Through Evolutionary Relationships (PANTHER) analysis of cellular component (**A**) and molecular function (**B**) of genes upregulated in RAS-STCs. (**C**) Database for Annotation, Visualization, and Integrated Discovery (DAVID) 6.8 functional annotation analysis of messenger RNAs upregulated in RAS-STCs.

**Figure 5:**
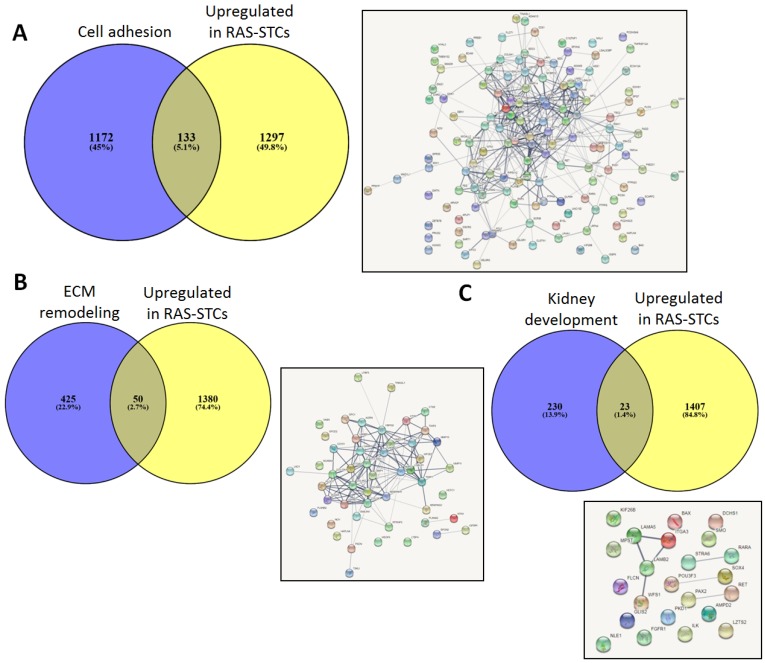
Venn diagram analysis (Venny 2.1) indicated that 133 cell adhesion (**A**) 50 extracellular matrix remodeling (**B**) and 23 kidney development (**C**) genes were upregulated in renal artery stenosis (RAS)-scattered tubular-like cells (STCs). Search Tool for the Retrieval of Interacting Genes (STRING) was used to predict associations between these genes.

Annotation analysis showed that genes upregulated in RAS-STCs (Log2 fold change > 1, *p* < 0.05) have an important cellular and organelle component (Figure 4A) with binding and catalytic activity (Figure 4B). Functional analysis revealed that RAS-STCs have a higher expression of genes encoding for proteins implicated in cell adhesion, extracellular matrix organization and remodeling, and kidney development (Figure 4C). Venn diagram analysis showed that 133 genes implicated in cell adhesion (Figure 5A), 50 involved in extracellular matrix remodeling (Figure 5B), and 23 in kidney development (Figure 5C) were upregulated in RAS-STCs.

### 2.5. Genes Downregulated in RAS-STCs

**Figure 6:**
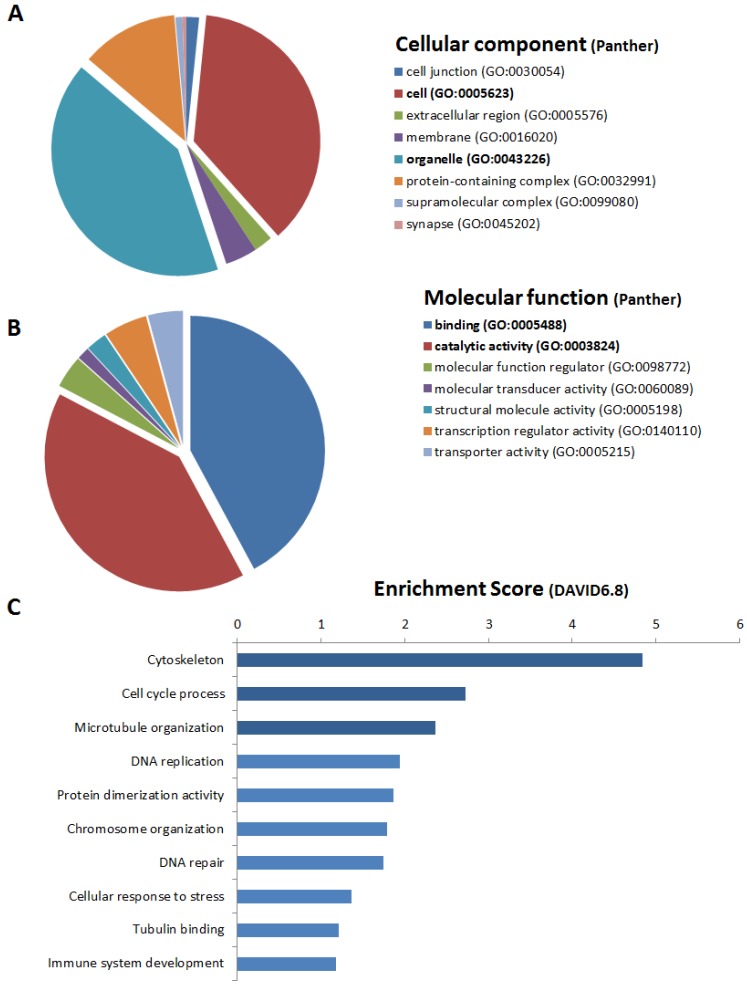
Genes downregulated in renal artery stenosis (RAS)-scattered tubular-like cells (STCs). Protein Analysis Through Evolutionary Relationships (PANTHER) analysis of cellular component (**A**) and molecular function (**B**) of genes downregulated in RAS-STCs. (**C**) Database for Annotation, Visualization, and Integrated Discovery (DAVID) 6.8 functional annotation analysis of messenger RNAs downregulated in RAS-STCs.

**Figure 7:**
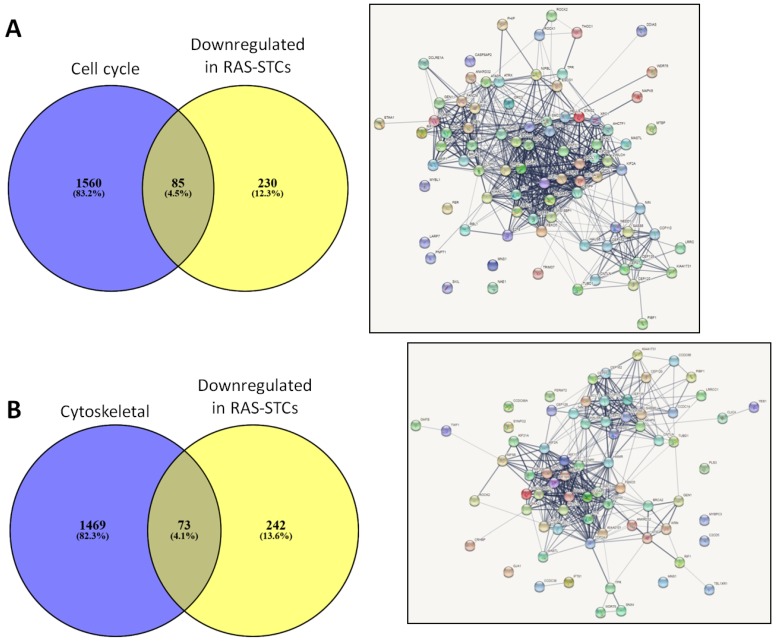
Venn diagram analysis (Venny 2.1) indicated that 85 cell cycle (**A**) and 73 cytoskeletal (**B**) genes were downregulated in RAS-STCs. Search Tool for the Retrieval of Interacting Genes (STRING) was used to predict associations between these genes.

Genes downregulated in RAS-STCs (Log2 fold change <−1, *p* < 0.05) have also primarily cellular and organelle components (Figure 6A) and possess binding and catalytic activity (Figure 6B). These genes encode for cell cycle and cytoskeletal proteins (Figure 6C). Venn diagram analysis indicated that 85 cell cycle genes (Figure 7A) and 73 cytoskeletal genes (Figure 7B) were downregulated in RAS-STCs. 

### 2.6. Validation of mRNAseq Analysis 

Six candidate genes were validated by quantitative-polymerase chain reaction (qPCR). Expression of selected candidate genes followed the same patterns as the mRNAseq findings, where Dachsous Cadherin-Related 1 (DCHS1), Olfactomedin 4 (OLFM4), and Pleckstrin Homology Like Domain Family A Member 3 (PHLDA3) were upregulated and Centromere Protein F (CENPF), Claspin (CLSPN), and RNA Binding Motif Protein 41 (RBM41) were downregulated in RAS-STCs (Figure 3B).

### 2.7. STC Activation and Proliferation

**Figure 8:**
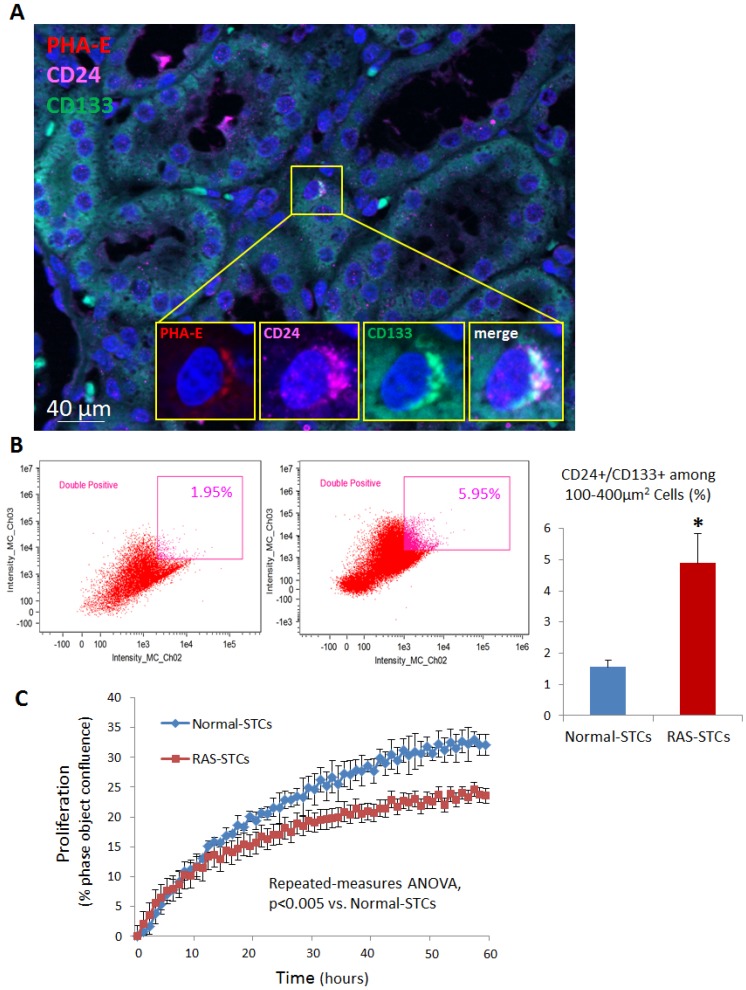
Scattered tubular-like cells (STC) activation and proliferation. (**A**) Representative immunofluorescence staining (40*X* and 100*X*) for the STC surface markers CD133 (green) and CD24 (pink), and the tubular cell marker phytohemagglutinin (PHA)-E (red) showing CD133+/CD24+ cells in renal proximal tubules. (**B**) Flow cytometry analysis showing that the percentage of CD24+/CD133+ STCs from dissociated kidney cells was higher in RAS versus Normal pigs (*n* = 3 each). (**C**) Cell proliferation, measured in percent phase object confluence unit, was impaired in RAS-STCs (Repeated-measures analysis of variance (ANOVA), *n* = 3 each). * *p* < 0.05 vs. Normal-STCs.

Immunohistological staining, with antibodies against CD133 and CD24 and the tubular cell marker phytohemagglutinin (PHA)-E, was performed to identify STCs in frozen pig kidney sections. CD133+/CD24+ cells were detected in proximal tubules (PHA-E) in situ (Figure 8A). STC activation was assessed by counting the total number of STCs in pig kidney sections using flow cytometry with CD133 and CD24 antibodies. The percentage of CD24+/CD133+ STCs among dissociated kidney cells was higher in RAS (4.90%) compared to Normal (1.56%) pigs (Figure 8B). Contrarily, STC proliferation rate decreased in RAS-STCs (Figure 8C, Repeated-measures analysis of variance (ANOVA), *p* < 0.05). 

## 3. Discussion

In the current study, we used a swine model that recapitulates human RAS to test the hypothesis that chronic renal ischemia and hypertension alter the gene expression profile of STCs. RAS is characterized by repeated episodes of insults mimicking AKI [13,14], and therefore represents a relevant model to study this endogenous repair mechanism. While ischemia-reperfusion injury activates STCs capable of repairing the damaged kidney, the effects of chronicity renal ischemia and hypertension on these cells remain largely unknown. To shed light into this, we comprehensively characterized the expression of mRNAs in STCs harvested from Normal and RAS pig kidneys. We identified a significant number of genes upregulated in RAS- versus Normal-STCs. These mRNAs encode for proteins involved in cell adhesion. Cells expressing the pentaspanning transmembrane glycoprotein CD133 [15], which characterize immature or dedifferentiated cells [16,17], often express cell adhesion molecules [18]. Upregulation of cell adhesion molecules in AKI may promote inflammatory cell infiltration into the interstitium of the kidney [19].

Also of note is the up-regulation of mRNAs implicated in extracellular matrix remodeling, like several genes encoding for collagen (e.g., *COL6A2*, *COL18A1*). STCs display a strong expression of structural proteins like collagen, which serve as an attachment system that anchors the basal membrane of the epithelium to the underlying extracellular matrix [15]. Speculatively, STCs exposed to the hostile micro-environment of RAS may require a firmer attachment to underlying extracellular structures, which is reflected in increased expression of extracellular matrix remodeling genes.

Renal ischemia also upregulated STC genes involved in kidney development, such as sex-determining region Y-box (*SOX*) genes. Previous studies have shown that in response to acute hypoxia or toxic injury, there is a rapid and robust proliferation of surviving epithelial cells, which is accompanied by the expression of *SOX* genes [20,21]. Therefore, RAS-induced expression of kidney development genes may represent the initial step of surviving tubular cells in response to injury.

Contrarily, genes downregulated in RAS-STCs are related to cell cycle, including genes implicated in the structural maintenance of chromosomes (e.g., *SMC2*, *SMC4*) and those with helicase-like activity (e.g., *BLM*, *HELLS*). Chromosomes undergo continuous morphological changes as cells advance through the cell cycle, which are in part orchestrated by *SMC* genes [22]. Likewise, helicases are essential during DNA replication [23]. Hence, downregulation of these genes could compromise cell division in RAS-STCs.

mRNAs encoding for cytoskeletal proteins were also downregulated in RAS-STCs. Expression of cytoskeletal proteins increases in regenerating, dividing STCs after ischemic injury [24], and may confer resilience against cellular insults [25]. Thus, downregulation of cytoskeletal genes may have a negative impact on the reparative capacity of STCs.

Notably, RAS-induced changes in the mRNA profile were associated with STC activation, reflected in an increased percentage of STCs from dissociated kidney cells. This is likely due to upregulation of genes implicated in kidney development, which might have facilitated the dedifferentiation process that activates STCs. This is supported by the previous observation that surviving tubular epithelial cells upregulate *SOX* genes before expressing kidney injury markers like neutrophil gelatinase-associated lipocalin or kidney injury molecule-1 [20].

However, cell proliferation was blunted in RAS-STCs, possibly due to impaired cell division and decreased expression of cytoskeletal proteins. Cell division is achieved by self-organized structures called mitotic spindles, which are formed by ensembles of cytoskeletal networks [26]. Similarly, loss of cytoskeletal proteins may lead to cell cycle arrest [27], underscoring their important role in cell proliferation. Therefore, our observations suggest that RAS-induced changes in the gene expression profile of STCs may diminish their potency and reparative capacity.

Our study is limited by the modest number of samples, although such sample sizes are often employed in sequencing studies [28,29,30]. Our RAS model uses relatively young pigs and the duration of the disease is shorter than in humans. Nevertheless, our animals displayed the main features of human RAS. Evidently, exposure to the effects of ischemia and hypertension for 10 weeks was sufficient to alter the genetic content and impair the function of STCs. Culture conditions may affect gene and protein expression of STCs but given that Normal- and RAS-STCs were cultured in a similar way, renal ischemia and hypertension could have significantly contributed to changes in the gene expression profile of RAS-STCs. Further studies are required to establish the genomic dysregulation on STC reparative properties. Future studies are also needed to confirm these findings and explore the longitudinal effects of RAS on STCs.

In summary, our study shows that RAS in swine modifies the mRNA expression profiles of STCs, upregulating genes involved in cell adhesion, extracellular matrix remodeling, and kidney development, but downregulating cell cycle and cytoskeleton genes. These findings were associated with increased STC activation and impaired proliferation. Therefore, our observations have important functional implications and suggest that RAS may limit the regenerative potential of STCs. Future studies are needed to characterize the gene expression profile of human STCs and develop novel interventions to enhance proliferation of STCs in subjects with RAS.

## 4. Materials and Methods 

### 4.1. Experimental Design 

All animal procedures were approved by the Institutional Animal Care and Use Committee (A00004330, approved on 4/9/2019, Rochester, MN, USA). Six female domestic pigs (Manthei Hog Farm, Elk River, MN, USA) were studied for 10 weeks. We opted for using female pigs to test whether the effects on hypertension and renal ischemia on STCs outweigh any gender-specific protection. At baseline, animals were anesthetized with intramuscular tiletamine hydrochloride/zolazepam hydrochloride (5 mg/kg, Telazol^®^, Fort Dodge Animal Health, New York, NY, USA) and xylazine (2 mg/kg), and anesthesia was then maintained with intravenous ketamine (0.2 mg/kg/min) and xylazine (0.03 mg/kg/min). Unilateral RAS was induced in 3 animals (RAS group) by placing a local irritant coil in the main renal artery using fluoroscopy (Siemens, Munich, Germany), which leads to the gradual development of unilateral RAS over a 7–10-day period, as reported previously [31]. The remaining 3 animals underwent a sham procedure (Normal group).

Ten weeks later, all animals were similarly anesthetized and the degree of stenosis was evaluated by angiography. Single-kidney hemodynamics and function were determined using MDCT. Briefly, renal regional perfusion, RBF and GFR were a Flash 128 MDCT scanner (Somatom Definition Flash, Siemens Healthcare, Erlangen, Germany), as previously described [32,33]. Following a bolus of iopamidol (0.5 cc/kg/2s), 70 multi-scan exposures were acquired at a cycle time of 0.67 s, and then by 70 scans at a cycle time of 2 s. Tissue time-attenuation curves obtained in regions of interest selected from the aorta, renal cortex, and medulla were fitted by curve-fitting algorithms to obtain measures of renal function. Cortical and medullary volumes were calculated by planimetry and images were analyzed with Analyze (Biomedical Imaging Resource, Mayo Clinic, Rochester, MN, USA). Single-kidney GFR was calculated from the cortical curve slope and RBF by multiplying kidney volume (mL of tissue) by renal perfusion (mL/min per mL of tissue). Blood pressure measured during MDCT studies with an intra-arterial catheter [32]. Systemic blood samples were collected for serum creatinine measurements (Gamma-Coat kit; DiaSorin, Stillwater, MN, USA).

A few days after MDCT studies, animals were euthanized with sodium pentobarbital (100 mg/kg IV, Fatal-Plus, Vortech Pharmaceuticals, Dearborn, MI, USA). The kidneys were removed using a retroperitoneal incision, immediately dissected. Kidney cells were then dissociated and the number of STCs quantified by flow cytometry. STCs were isolated from fresh pig kidneys, cultured, characterized, and RNA isolated for mRNA sequencing analysis.

### 4.2. STC Isolation and Characterization

STCs were isolated from fresh pig kidneys, as previously shown [7,12]. Briefly, pig cortical and medullary sections were washed with phosphate-buffered saline, diced, and digested with 2 mg/mL collagenase for 1 h. Samples were then forced through a 60-mesh (250 μm) steel sieve to remove the fibrous component [34]. The cellular fraction was passed through 100 μm cell strainer followed by the addition of Medium 199 containing 3% FBS (Gibco BRL, Waltham, MA, USA) [35] at 37 °C in a humidified atmosphere with 5% CO2. The culture medium was replaced every 2 days to remove non-adherent cells. Two weeks later, the adherent cells were harvested with TrypLE™ Express (Gibco) treatment and sub-cultured. Cultured pig STCs were characterized using flow cytometry and immunofluorescence staining, which confirmed their positivity for CD133 (Novus Biologicals, Centennial, CO, USA) and CD24 (Abcam, San Francisco, CA, USA).

### 4.3. mRNAseq and Bioinformatic Analysis

mRNA sequencing analysis was performed as previously described [36]. Sequencing RNA libraries were prepared following the manufacturer’s protocol (TruSeq RNA Sample Prep Kit v2, Illumina) and loaded onto flow cells (8–10 pM) to generate cluster densities (700,000/mm^2^, Illumina cBot and cBot Paired-end cluster kit). STCs were sequenced on an Illumina HiSeq 2000 using TruSeq SBS kit version-3 and HCS v2.0.12 data collection software [37,38,39].

Raw Fastq files were processed through Mayo’s internal MAP-RSeq pipeline [37] (Version 3.0). MAP-RSeq uses a variety of publically available bioinformatics tools tailored by in-house developed methods. The aligning and mapping of reads were performed using Star aligner against the pig reference genome (Sus scrofa 11.1) [40]. The gene and exon counts were generated by FeatureCounts using the gene definitions files from Ensembl v11.1.96 [39]. Quality control was carried out using RSeqQC [41] and some additional metrics [42] to ensure the results from each sample were reliable and could be collectively used for differential expression analysis. Differential expression analysis was carried out using edgeR [43] (version 3.20.1). Differentially expressed genes were identified with average expression larger than 2 CPM, absolute log2 fold change larger than 1, and adjusted *p*-value smaller than 0.05 (R software, version 3.5.2). Genes up-regulated and down-regulated in RAS-STCs were classified by their cellular component and molecular function using Protein Analysis Through Evolutionary Relationships (PANTHER) [44]. The Database for Annotation, Visualization, and Integrated Discovery (DAVID6.8, http://david.abcc.ncifcrf.gov/) [45,46] was used to obtain a ranking of primary gene ontology categories for genes up-regulated and down-regulated in RAS-STCs. Two-way Venn diagrams were constructed using VENNY 2.1 (http://bioinfogp.cnb.csic.es/tools/venny/) to visualize the total number genes upregulated or downregulated in the top functional category, whereas Search Tool for the Retrieval of Interacting Genes (STRING) version 9.1 (http://string-db.org/) was used to predict associations between these genes.

### 4.4. Validation of mRNAseq Analysis

RNA isolation, cDNA synthesis, and qPCR using the ΔΔCt method were used to randomly evaluate gene expression levels of *DCHS1, OLFM4, PHLDA3, CENPF, CLSPN*, and *RBM41* (ThermoFisher Scientific (*CENPF*: ss06882664, *CLSPN*: ss06901525, *DCHS1*: ss06912739, *OLFM4*: ss06887455, *RBM41*: *APU66GY*, and *PHLDA3*: APPRNR7) in Normal- and RAS-STCs. Gene expression was normalized to GAPDH.

### 4.5. STCs Activation

STC activation was assessed by counting the total number of STCs in pig kidney sections using flow cytometry (FlowSight, Amnis, Seattle, WA, USA) with CD133 (1:25, Novus Biologics, Centennial, CO, USA cat#: NB120-16518G) CD24 (1:25, Abcam, cat#: ab134375) antibodies [12]. At least 100,000 events were acquired. Following particle focusing, doublets, cell clusters, and debris were eliminated by gating on events with a high aspect ratio (>0.6) and for cell size (10–20 µm diameter, 100 to 400 µm^2^ area) using bright field area vs. aspect ratio features. CD133 and CD24 positive events were quantified using flow-gaiting strategy: number of double-positive CD133 and CD24 events/number of singlet events between 10–20 µm diameter and expressed as a percentage. To detect STCs in frozen pig kidney sections, we performed double-staining of CD133 (1:100, Novus Biologicals, Centennial, CO, USA) and CD24 (1:100, Abcam, San Francisco, CA, USA), and the tubular marker phaseolus vulgaris erythroagglutinin (PHA-E, Invitrogen, Carlsbad, CA, USA) [12].

### 4.6. STC Proliferation

To assess cell proliferation, STCs were harvested in M199 media with 3% FBS and 2500 cells/well seeded onto a 96-well tissue-culture plate. Cell confluence was imaged in real-time by phase-contrast using the IncuCyte Zoom (Essen Biosciences Inc., Ann Arbor, MI, USA) system30, 31. Frames were captured at 2-h intervals from 60 h, analyzed using the integrated confluence algorithm, and proliferation growth curves constructed using IncuCyte™ Zoom software.

### 4.7. Statistical Analysis

Statistical analysis was performed using the JMP Pro 14.0 software (SAS Institute, Cary, NC, USA). Results are expressed as means ± SD. Comparisons among groups were performed using Student’s *t*-test. Statistical significance for all tests was accepted for *p* < 0.05. Repeated-measures ANOVA, which takes measurements on the same experimental unit over time, taking into account the probability that measurements for a given experimental unit will be correlated in some way [47], was used to compare cell proliferation between Normal- and RAS-STCs.

## Figures and Tables

**Table 1:** Systemic characteristics and renal function at 10 weeks.

Parameter	Normal	RAS
Body Weight (Kg)	50.3 ± 0.6	54.3 ± 5.5
Degree of stenosis (%)	0	84.7 ± 12.7 *
Systolic blood pressure (mmHg)	96.3 ± 5.1	151.7 ± 33.3 *
Diastolic blood pressure (mmHg)	70.0 ± 6.0	118.7 ± 21.7 *
Mean arterial pressure (mmHg)	78.8 ± 5.3	129.7 ± 25.5 *
Serum creatinine (mg/dL)	1.2 ± 0.1	1.8 ± 0.3 *
Cortical volume (mL)	104.8 ± 4.4	58.7 ± 27.1 *
Medullary volume (mL)	19.6 ± 1.0	19.5 ± 2.3
Cortical perfusion (mL/min/mL tissue)	5.4 ± 0.4	2.4 ± 0.5 *
Medullary perfusion (mL/min/mL tissue)	2.9 ± 0.3	2.7 ± 0.3
RBF (mL/min)	607.4 ± 42.4	315.5 ± 94.4 *
GFR (mL/min)	98.1 ± 9.5	52.2 ± 8.3 *

* *p* ≤ 0.05 vs. Normal.

## Supplementary Materials

The following are available online at https://www.mdpi.com/1422-0067/20/20/5069/s1.

## Abbreviation

AKI	Acute kidney injury
STCs	Scattered tubular-like cells
RAS	Renal artery stenosis
RBF	Renal blood flow
mRNAseq	Messenger RNA sequencing
MDCT	Multi-detector computed tomography
GFR	Glomerular filtration rate
CPM	Counts per million
qPCR	Quantitative-polymerase chain reaction
BCAM	Linear dichroism basal cell adhesion molecule
CADH3	Cadherin 3
CADH16	Cadherin 6
EpCAM	Epithelial cellular adhesion molecule
COL6A2	Collagen 6A2
COL18A1	Collagen 18A1
LAMA5	Laminin A5
LAMB3	Laminin B3
POU3F3	POU Class 3 Homeobox 3
SOX	Sex determining region Y-box
PANTHER	Protein Analysis Through Evolutionary Relationships
DAVID	Database for Annotation, Visualization, and Integrated Discovery
STRING	Search Tool for the Retrieval of Interacting Genes
PHA-E	Phaseolus vulgaris erythroagglutinin
